# Impact of Physical Therapy on Empowering Neurological Aging: A Narrative Review

**DOI:** 10.7759/cureus.95640

**Published:** 2025-10-29

**Authors:** Maheshkumar Baladaniya, Shraddha Baldania, Nensi V Gandhi, Anamitra Hait

**Affiliations:** 1 Department of Physical Therapy and Rehabilitation, Neighborhood Physical Therapy PC, New York, USA; 2 Department of Physical Therapy, Enjoy Rehab PT PC, New York, USA; 3 College of Physiotherapy, Sumandeep Vidyapeeth Deemed to be University, Vadodara, IND; 4 Department of Medicine, KG Hospital, Chittaranjan, IND

**Keywords:** daily living activities, health aging, neurological diseases, physical therapy rehabilitation, rehabilitation telerehabilitation

## Abstract

Aging is an inevitable biological process that is frequently accompanied by neurological decline, which profoundly impacts gait, balance, and the ability to perform activities of daily living. Physical therapy (PT) plays a pivotal role in mitigating these deficits by enhancing mobility, strength, and independence through evidence-based interventions like resistance training, balance exercises, and functional mobility programs. This narrative review synthesizes current evidence on PT’s effectiveness in managing age-related neurological changes, emphasizing its integration within interdisciplinary teams and the use of innovative technologies such as exoskeletons, telerehabilitation, and brain-computer interfaces. A combination of specific keywords and Boolean operators was utilized to identify peer-reviewed studies on the databases PubMed, Google Scholar, and ScienceDirect, focusing on the impact of PT in empowering neurological aging. PT encourages active engagement and enhances the quality of life for elderly people with neurological disorders. With an aging population, the demand for PT services is expected to continue rising, underscoring the importance of adequate resources and specialized training programs in this profession. Despite robust evidence supporting the benefits of PT, gaps persist in understanding its long-term efficacy, optimal intervention dosing, and the integration of emerging technologies into routine practice. Challenges such as limited access to specialized services and insufficient data on cost-effectiveness and patient adherence further complicate the delivery of care. This review advocates for future research to refine PT strategies, enhance interdisciplinary collaboration, and leverage technological advancements to optimize outcomes for older adults with neurological conditions, ultimately promoting successful aging and sustained independence.

## Introduction and background

Aging is an inevitable process often accompanied by neurological decline. Epidemiological data reveal that 35% of community-dwelling adults over 70 years exhibit abnormal gait patterns, while 32.2% of elderly individuals aged 60-97 years present with impaired gait, with the prevalence escalating significantly with advancing age [1,2]. This profoundly impacts balance and the ability to perform activities of daily living (ADL), thereby contributing to reduced mobility, increased fall risk, and diminished independence, posing substantial challenges to the quality of life (QOL) in aging populations. However, emerging evidence suggests that structured exercise interventions, particularly resistance and multicomponent programs, offer promising benefits. Recent systematic reviews highlight that such programs enhance muscle strength, improve gait, and boost functional capacity in older adults with sarcopenia and mobility limitations, providing a foundation for mitigating the physical consequences of neurological aging [3]. According to the World Health Organization’s latest Global Status Report on Neurology, these age-related neurological conditions now affect over 42% of the global population (more than 3.4 billion people) and are responsible for 11.8 million deaths annually, highlighting the urgent need for scalable interventions to address mobility limitations and sarcopenia in aging populations [4].

The landscape of rehabilitation is evolving with technological advancements that expand therapeutic options for clinicians. Innovations such as mixed reality and exoskeleton-assisted training have demonstrated encouraging early results in restoring walking performance and task-specific mobility, offering tailored support for individuals with neurological impairments [5]. Additionally, for improving balance and gait outcomes across both older adults and those with neurological conditions, telerehabilitation and interactive remote platforms have proven effective, facilitating higher-dose, task-specific practice in home and community settings [6]. These developments enhance the reach and efficacy of rehabilitation efforts, underscoring a shift toward more accessible and personalized care.

Physical therapy (PT) plays a multifaceted role in addressing the challenges of neurological aging, emerging as a cornerstone within an interdisciplinary team. Intensive PT in acute care settings is particularly critical, focusing primarily on restoring musculoskeletal and neurological function. PT addresses secondary complications such as frailty, weariness, sarcopenia, and falls, which are prevalent among the elderly [7,8]. The main aim of the PT is to restore lost capabilities, prevent functional decline, and maintain long-term independence in ADL, which includes precise limb positioning and the management of hypertonic or spastic muscles, which are common in conditions like stroke or Parkinson’s disease, thereby supporting early recovery and preventing further deterioration [8]. Hence, PT integration into holistic care demonstrates its ability to meet the varied needs of aging individuals with neurological changes [7,8].

Current literature provides useful evidence for specific interventions, such as resistance exercises and gait training, yet the optimal dosing, long-term efficacy, and integration of emerging technologies like telerehabilitation and exoskeletons into routine practice remain underexplored. Moreover, limited standardized protocols for diverse neurological disorders, inconsistencies in access to specialized PT services, and a lack of comprehensive data on cost-effectiveness and patient adherence further complicate care delivery. Hence, this narrative review is necessitated to synthesize existing evidence, identify these research gaps, and advocate for future studies that can refine PT strategies, enhance interdisciplinary collaboration, and improve outcomes for older adults aging with neurological changes.

## Review

**Figure 1:**
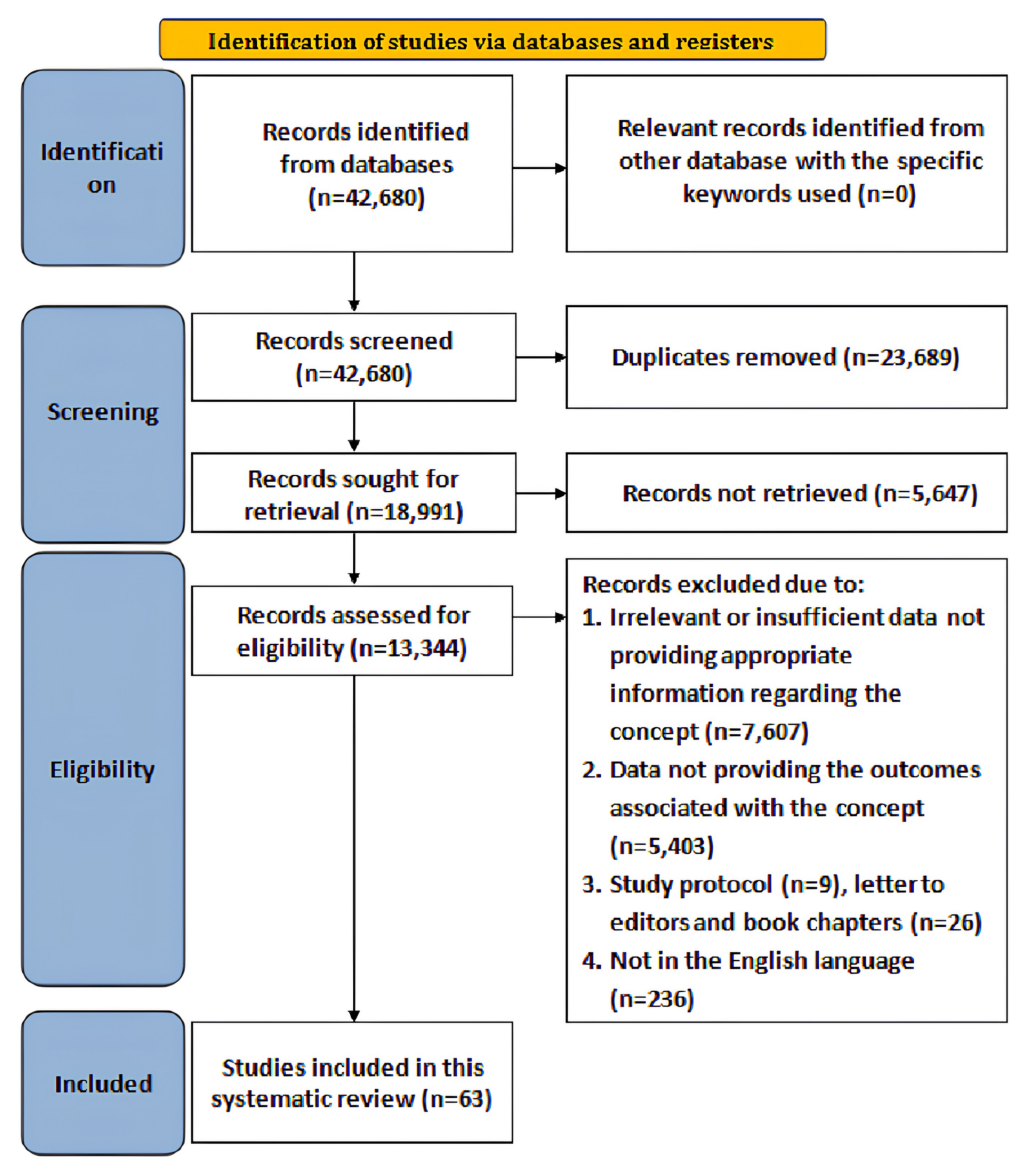
Search strategy

The Preferred Reporting Items for Systematic reviews and Meta-Analyses (PRISMA) framework [9], as illustrated in Figure 1, and the Scale for the Assessment of Narrative Review Articles (SANRA) [10] guided article selection, inclusion/exclusion criteria, critical appraisal, and evidence presentation.

Data sources and search strategy

A thorough literature search incorporating keywords with Boolean operators that consisted of ((((Physical therapy OR Physiotherapy)) AND (Aging OR Neurological changes OR Neurological disorders)) AND (Patient outcome assessment OR Treatment outcome evaluation)) was conducted on PubMed, ScienceDirect, and Google Scholar databases from 2010 to August 2025.

Study screening and selection

The inclusion criteria involved studies that concentrated on evaluating the role of PT interventions at any phase of aging with neurological changes, including PT in assessment, treatment planning, and interventions for neurological conditions in the elderly, systematic reviews, meta-analyses, research articles assessing functional outcomes, QOL, gait, balance, mobility, or psychological benefits associated with PT in aging populations, peer-reviewed articles, editorials, conceptual articles, and published reports, open access studies published in English. However, investigations that focused solely on pharmacological or surgical interventions without reference to PT, studies lacking an available English translation or not published in English, with only a title and no abstract, those without open access, and those lacking sufficient knowledge with respect to the context were excluded.

Four reviewers independently assessed article eligibility for inclusion. To remove duplicates, titles and abstracts were screened initially, followed by a second review to exclude ineligible studies and a full-text assessment to confirm inclusion. Reviewer disagreements were resolved through discussion and consensus. To synthesize the findings, a critical narrative approach was employed. The studies involved diverse methodologies and outcome measures, leading to substantial heterogeneity.

Data extraction and synthesis

The data was extracted based on the information that concentrated on evaluating the role of PT interventions in aging with neurological changes, including PT in assessment, treatment planning, and interventions for neurological conditions in the elderly, and research articles assessing functional outcomes, QOL, gait, balance, mobility, or psychological benefits associated with PT in aging populations. The critical narrative technique was utilized to incorporate figures and text to summarize and validate evidence.

Neurological age-related changes

Normal aging is characterized by a progressive reduction in regional brain volume, with notable effects on critical areas such as the prefrontal cortex, hippocampus, and cerebellum [11]. Research indicates that hippocampal volume loss accelerates particularly around the fifth decade of life, though executive function and memory may remain relatively intact in healthy aging individuals [11]. This age-related neurological impairment arises from a complex interplay of factors, including alterations in neurotransmitter activity, neuronal loss, accumulation of toxic chemicals, and genetic variations, all of which contribute to the gradual decline in brain health [12].

Furthermore, these changes can manifest as reduced neuroplasticity, impacting cognitive reserve and increasing vulnerability to neurodegenerative processes. For instance, evidence suggests that age-related declines in synaptic density and myelin integrity further exacerbate cognitive and motor deficits, with studies showing up to 20-30% loss in white matter volume by age 80 [12,13]. PT interventions, such as targeted exercise programs, can promote neuroplasticity by stimulating the expression of brain-derived neurotrophic factor (BDNF), potentially mitigating these volumetric losses and supporting functional brain adaptations. This underscores the preventive role of PT in maintaining neural integrity during aging [13].

Anatomically, key changes include brain shrinkage, particularly in the frontal cortex, alongside a progressive loss of nerve cells in both the brain and spinal cord [12]. Functionally, these alterations manifest early as deficits in short-term memory and new learning capacity, while verbal abilities and intellectual performance tend to remain preserved in the absence of underlying neurological or vascular pathologies [12]. The varied impact of cognitive aging underscores its complexity, paving the way for targeted therapies to reduce functional decline.

Pathophysiological mechanisms in age-related neurological disorders

Age-related neurological disorders involve complex pathophysiological mechanisms that contribute to progressive functional decline [14]. These mechanisms include protein misfolding and aggregation (amyloid-β plaques and tau tangles in Alzheimer’s disease and α-synuclein aggregates in Parkinson’s disease), oxidative stress and mitochondrial dysfunction, neuroinflammation, vascular insufficiency leading to ischemic damage, autoimmune-mediated demyelination (multiple sclerosis, MS), and progressive motor neuron degeneration (amyotrophic lateral sclerosis) [14,15]. Understanding these underlying pathophysiological processes is essential for developing targeted PT interventions that can address specific impairments, promote neuroplasticity, and optimize functional recovery in elderly patients with neurological conditions [14,15].

In addition, chronic low-grade inflammation (“inflammaging”) plays a pivotal role, accelerating neuronal apoptosis and impairing synaptic function, which can be partially counteracted through PT-induced anti-inflammatory effects via improved circulation and muscle-derived myokines [16]. For example, resistance training has been shown to reduce oxidative stress markers in older adults, enhancing mitochondrial efficiency and potentially delaying disease progression in conditions like Parkinson’s, where dopaminergic neuron loss is exacerbated by free radical damage. These mechanisms highlight how PT can intervene at the cellular level to support neuronal survival and repair [16].

Neurological conditions in elderly populations

Parkinson’s disease is a progressive neurodegenerative disorder primarily resulting from the degeneration of dopaminergic neurons in the substantia nigra, characterized by alpha-synuclein protein aggregation in brain cells. This neuronal loss leads to the classic motor symptoms, including tremors, muscle rigidity, bradykinesia, postural instability, and freezing episodes, alongside non-motor symptoms such as cognitive changes and autonomic dysfunction [17]. Recent evidence indicates that PT interventions like rhythmic auditory stimulation can improve gait freezing by enhancing basal ganglia function, with studies reporting up to 25% improvement in stride length and reduced fall risk in moderate-stage patients [18]. Alzheimer’s disease and dementia are progressive neurocognitive disorders characterized by memory loss, impaired executive function, language disturbances, and behavioral changes, including agitation, depression, and sleep disturbances. The condition progresses through mild, moderate, and severe stages, with each phase presenting distinct PT challenges and intervention opportunities [19]. Multicomponent PT programs combining aerobic and cognitive exercises have demonstrated moderate effects on slowing cognitive decline, with meta-analyses showing improvements in Mini-Mental State Examination scores by two to three points over six months [20].

Stroke is a cerebrovascular accident resulting from an interrupted blood supply to brain tissue, causing neuronal death and functional deficits. Strokes are classified into two main categories: ischemic strokes (85% of cases), caused by thrombotic or embolic arterial occlusion, and hemorrhagic strokes (15% of cases), resulting from intracerebral or subarachnoid hemorrhage. Both types present similar rehabilitation challenges, including hemiparesis, balance impairments, and functional mobility deficits, though acute management and prognosis may differ significantly [21]. Constraint-induced movement therapy, an evidence-based PT approach, has been effective in restoring upper limb function post-stroke, with randomized trials showing 20-30% gains in motor scores when applied early in recovery [22].

Spinal disorders, including degenerative spondylosis, spinal stenosis, and osteoporosis-related vertebral fractures, are common in the elderly due to age-related degeneration of the spine. These conditions cause chronic back pain, radiculopathy, and restricted mobility, with severe cases leading to neurological deficits like myelopathy. PT emphasizes pain management, core strengthening, and posture training to improve spinal stability and functional movement [23]. Aquatic therapy has emerged as a supportive intervention, reducing compressive forces on the spine and improving pain scores in older adults with stenosis [24]. Seizure disorders, including late-onset epilepsy, often arise in older adults secondary to stroke, neurodegenerative diseases, or metabolic imbalances. These disorders manifest as recurrent seizures, which may cause falls, injuries, or post-ictal confusion, impacting functional independence. PT interventions include balance and coordination training to mitigate fall risk and promote safety during daily activities [25].

ALS is a progressive neurodegenerative condition affecting motor neurons in the brain and spinal cord, leading to gradual muscle weakness and coordination difficulties that progressively impair physical function and independence [26]. Respiratory-focused PT, such as inspiratory muscle training, can extend ventilator-free survival by several months, with studies emphasizing early intervention to preserve bulbar function [27]. MS, an autoimmune disorder, targets the central nervous system by damaging myelin sheaths surrounding nerve fibers in the brain, spinal cord, and optic nerves. This demyelination disrupts nerve impulse transmission, resulting in a spectrum of motor, sensory, and cognitive symptoms, including muscle weakness, coordination challenges, visual disturbances, and fatigue, which require adaptive therapeutic strategies [28]. High-intensity interval training adapted for MS has been shown to reduce fatigue by 15-20% and improve walking endurance, highlighting PT’s role in symptom management [29].

**Figure 2:**
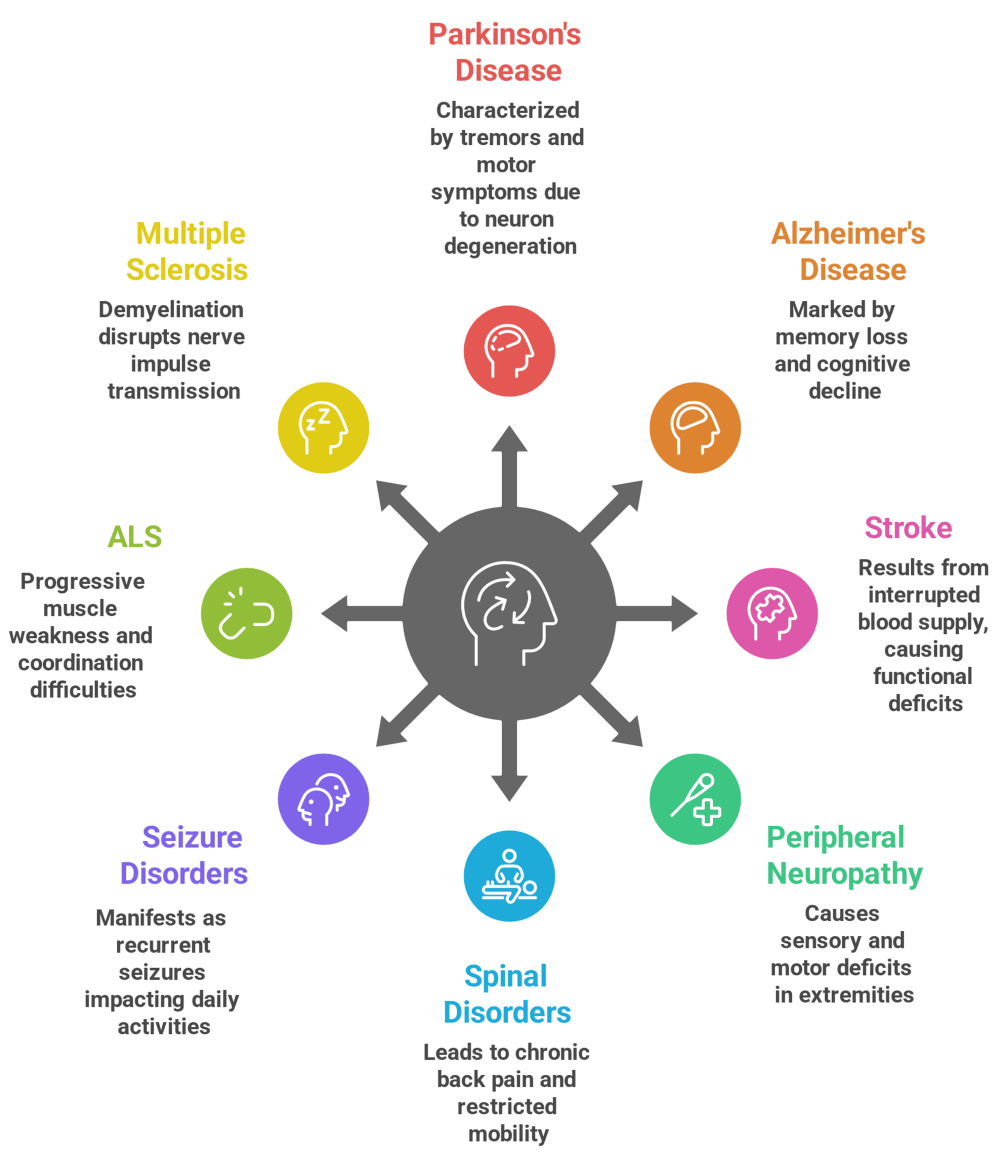
Neurological conditions in the elderly population ALS, amyotrophic lateral sclerosis

Peripheral nervous system disorders, such as peripheral neuropathy, are prevalent in older adults, often resulting from diabetes, vitamin deficiencies, or idiopathic causes. These disorders commonly present with sensory symptoms like numbness, tingling, or burning pain in the extremities, and motor deficits that impair balance and gait, increasing fall risk. PT interventions focus on sensory reeducation, strength training, and balance exercises to enhance mobility and reduce fall incidence [30]. The various conditions are illustrated in Figure 2 [17-30].

Complex role of PT

PT effectively targets movement impairments associated with neurological conditions, empowering individuals to enhance their functional capacity and regain independence. With nearly 100 million Americans affected by neurological disorders, including migraine, stroke, and dementia, the demand for specialized neurological PT continues to rise, reflecting the growing need for expert care in this field [31]. Rising demand is projected from epidemiological trends showing gait disorder prevalence escalating from 10% in those aged 60-69 to over 60% beyond 80 [2,32,33], and Alzheimer’s disease affecting 10% post-65 rising to 40% post-85, with global dementia cases projected to quadruple to 150 million by 2050 amid aging demographics [19,20] directly tying to heightened need for neuro-PT expertise in functional restoration for ADL/mobility in this demographic.

Physical therapists specializing in neurorehabilitation are equipped with advanced degrees in PT, emphasizing a neurological focus, alongside extensive clinical experience or completion of neurological residency or fellowship programs. Many also pursue board certification in neurologic PT (Neurologic Clinical Specialist) to affirm their expertise [12,14]. These professionals maintain proficiency through ongoing continuing education and demonstrate comprehensive skills in neurological assessments, evaluating aspects such as strength, range of motion, muscle tone, coordination, balance, gait, and sensory function.

Neurological physical therapists craft individualized treatment plans, integrating evidence-based interventions such as neuromuscular reeducation, therapeutic exercise, functional training, balance training, gait training, and the prescription of assistive devices. These approaches are meticulously tailored to address each patient’s specific impairments, align with their functional goals, and adapt to the progression of their neurological condition [34]. Beyond core interventions, PT incorporates patient education on self-management strategies, which have been linked to better long-term adherence and reduced healthcare utilization in aging populations with chronic neurological issues [35].

Gait and mobility improvement

Balance and mobility impairments significantly affect older adults, with 13% of individuals aged 65-69 years reporting imbalance, a figure that rises to 46% among those aged 85 years and older. The prevalence of gait problems among community-dwelling older adults aged 70 years and above reaches 35%, and abnormal gait is linked to a 2.2-fold increased risk of institutionalization and mortality compared to their peers without such disorders [35,36].

Aging is associated with a decline in balance, evidenced by increased postural sway, particularly when visual input is removed. Older individuals exhibit faster sway with their eyes closed, suggesting deterioration of the vestibulocochlear system. Age-related changes also encompass inappropriate activation of antagonist muscles during balance tasks and the adoption of altered postural strategies, characterized by enhanced hip flexion and extension movements [36,37].

Common gait patterns in older adults are often linked to conditions such as polyneuropathy, bilateral vestibulopathy, visual impairment, parkinsonism, cerebellar ataxia, and dementing syndromes. Frontal gait disorders are characterized by slower walking speed, shorter stride length, reduced cadence, a wider support base, and prolonged double-support phases. Additionally, many older adults adopt a “cautious” gait pattern, marked by decreased walking speed, reduced step length, and increased step timing variability [32,33].

Traditional balance and gait training encompasses progressive exercises designed to enhance static and dynamic balance, weight shifting, stepping patterns, and functional mobility tasks. Walking programs specifically target hip flexor contractures, a common gait impediment in the elderly population, aiming to restore more natural movement patterns [38]. Virtual reality-based training systems, such as the Balance Rehabilitation Unit, have shown significant improvements in balance parameters, with benefits sustained at a nine-month follow-up compared to standard fall prevention recommendations alone, highlighting the potential of technology-enhanced interventions [39]. Combined cognitive and physical training interventions yield superior outcomes compared to physical training alone, demonstrating sustained improvements in gait variables over six-month training periods among older adults with preserved cognitive function, suggesting a synergistic effect of dual-task approaches [40]. Structured balance and gait training protocols integrate progressive challenges, including static and dynamic balance exercises, functional stepping and weight-shifting activities, resistance training to strengthen lower extremities, and dual-task activities that blend cognitive and motor challenges [41]. These evidence-based interventions have proven effective in reducing fall risk, enhancing functional mobility, and improving QOL in older adults with neurological conditions. To further illustrate, sensor-based interventions providing biofeedback have demonstrated moderate to large effects on gait speed (0.5-1.0 m/s improvements) and balance scores in mixed elderly populations, with randomized trials emphasizing their superiority over conventional therapy for sustained gains [42]. These advancements reinforce PT’s efficacy in countering age-related gait deterioration through multifaceted, evidence-driven strategies.

Muscle strength and functional improvement

The decline in muscle strength with advancing age significantly compromises functional capacities essential for daily activities, including transfers, stair climbing, and general mobility [43]. This deterioration is influenced by multiple factors, such as sarcopenia, limited physical activity, joint dysfunction, and the presence of chronic diseases, which collectively exacerbate functional limitations in older adults [43]. Robust evidence from recent meta-analyses underscores the efficacy of resistance training in elderly individuals with neurological conditions [43]. A 2021 systematic review of 14 randomized controlled trials involving 561 older adults with sarcopenia revealed that resistance training significantly enhanced handgrip strength (SMD = 0.81, 95% CI 0.35 to 1.27, p = 0.0005), knee extension strength (SMD = 1.26, 95% CI 0.72 to 1.80, p < 0.0001), gait speed (SMD = 1.28, 95% CI 0.36 to 2.19, p = 0.006), and Timed Up-and-Go test performance (SMD = −0.93, 95% CI −1.30 to −0.56, p < 0.0001) compared to control groups [43]. Furthermore, long-term randomized trials incorporating resistance, balance, and cognitive-motor training components have demonstrated additional functional benefits [44]. Moreover, a six-month multicomponent program that combined physical and cognitive training, in comparison to physical training alone, found significantly reduced fall frequency by 77% (p < 0.001), improved gait parameters (p < 0.05), and enhanced functional fitness (p < 0.05) in community-dwelling older adults [44].

PT utilizes a diverse array of exercise modalities, including progressive resistance training to strengthen targeted muscles, functional movement training to develop activity-specific skills, neuromuscular reeducation to optimize motor patterns, and combined balance and coordination training with strengthening exercises to address multifaceted impairments [32]. The evidence indicates that approaches involving strength- and power-specific training contribute to improved functional outcomes, with power training showing particular benefits for rapid force production required in fall prevention and functional mobility tasks [45]. Moreover, functional training approaches incorporate task-specific movements that combine strength, balance, coordination, and endurance training within movement patterns relevant to the individual’s functional goals, directly translating improved performance in ADL [46].

Hence, applications of these approaches report considerable benefits across various neurological conditions [32], as for individuals with Alzheimer’s disease and dementia, progressive balance training combined with dual-task gait exercises has been shown to improve gait speed and reduce fall incidence over six months [44]. Moreover, a previous study reported significant enhancements in the dual-task cost of step time variability compared to a control group, underscoring the effectiveness of these interventions in managing mobility challenges associated with these disorders [44]. Additionally, in patients with Parkinson’s disease, high-intensity resistance and power training have led to increased motor control and improved sit-to-stand performance scores [45]. Papa et al. highlighted the potential of these targeted exercises to alleviate the motor symptoms characteristic of this condition by documenting huge, clinically useful gains in lower-limb power and functional mobility [45].

In ALS, respiratory muscle strengthening paired with posture training has proven advantageous in increasing peak cough flow and reducing dyspnea [46]. A study by Dehaghani et al. suggested that these interventions support respiratory function and overall QOL in ALS patients [46]. Individuals with MS have responded positively to combined resistance and balance circuit training, which correlates with reduced Timed Up-and-Go times and diminished fear of falling [47]. Kalron et al. observed consistent improvements in functional mobility after 12 weeks, indicating that this approach effectively enhances stability and movement confidence in this population [47].

Furthermore, multimodal exercise programs integrating strength, balance, and endurance training have successfully improved independence in ADL and QOL scores in older adults experiencing age-related functional decline. Additionally, benefits across mobility and self-care domains, emphasizing the comprehensive impact of these programs on preserving functional capacity in aging populations, were observed [48]. Building on this, heavy resistance training in older adults has been associated with neurometabolic changes, such as increased GABA levels in the brain, correlating with 10-20% strength gains and improved cognitive function after 12 weeks [49]. Long-term studies also indicate that one year of heavy resistance training at retirement age can preserve muscle function for up to four years, with sustained benefits in leg press strength and reduced sarcopenic progression. These findings emphasize PT’s role in inducing lasting neuromuscular adaptations [50].

Teamwork across disciplines

Occupational therapists complement PT by focusing on ADL that involve dressing, eating, personal hygiene, tailoring interventions to accommodate physical and cognitive limitations, cognitive adaptation (improving memory, problem-solving, and attention through structured routines and compensatory techniques) [17,51], and environmental modifications to promote independence in older adults with degenerative neurological diseases [51]. Occupational therapists additionally assess and modify environmental factors such as adjusting home layouts, installing assistive devices like grab bars, or improving lighting to reduce hazards and facilitate safer, more accessible living spaces [51]. This overall approach alleviates caregiver burden, thereby supporting functional autonomy and fostering a supportive ecosystem that enhances the overall QOL for individuals with progressive neurological conditions [51].

Neurological conditions such as stroke, Parkinson’s disease, MS, and ALS can significantly disrupt speech and swallowing functions through diverse mechanisms [52]. Cerebrovascular injury often impairs language processing, articulation, and swallowing by disrupting neural networks, compromising facial and throat muscle control, and causing hypophonic speech with reduced volume. It also affects neuronal transmission, leading to inconsistent muscle activation, rigidity, bradykinesia, and coordination difficulties [52-54]. In contrast, ALS involves the progressive loss of motor neurons that regulate these functions, culminating in severe and worsening speech and swallowing deficits over time [55].

Speech-language pathologists address these impairments with targeted interventions that involve tailored exercises to strengthen oral and pharyngeal muscles, techniques to improve articulation and voice projection, and strategies to ensure safe swallowing, offering complementary support that enhances overall functional outcomes when integrated with PT approaches to optimize rehabilitation and QOL for affected individuals [56].

Furthermore, physical therapists perform targeted respiratory muscle strengthening exercises to enhance lung capacity, postural training to optimize breathing mechanics, and coordination exercises that synchronize respiration with functional activities, thereby improving overall respiratory efficiency to address respiratory muscle weakness and dysfunctional breathing patterns, which can undermine vocal support and airway protection during swallowing, posing significant challenges for individuals with neurological conditions [46,57]. This interdisciplinary approach fosters comprehensive care by addressing the multifaceted needs of individuals with neurological diseases, integrating diverse therapeutic strategies to maximize functional potential and elevate QOL through collaborative intervention efforts.

**Figure 3:**
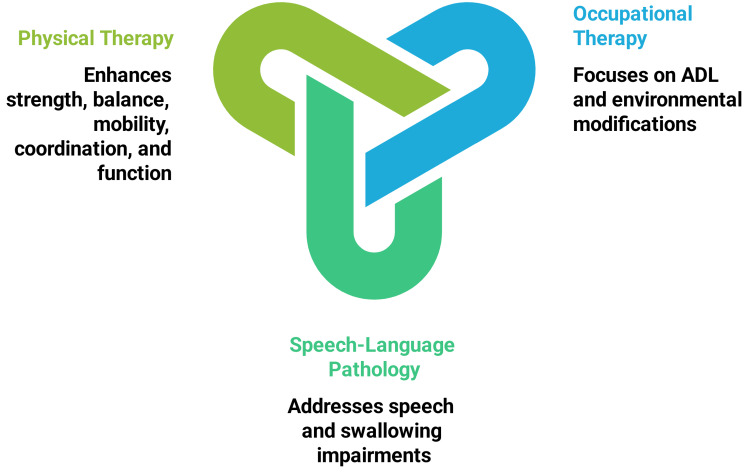
Interdisciplinary team collaboration for neurological rehabilitation ADL, activities of daily living

Interdisciplinary teams have been shown to reduce hospital readmissions by 15-25% in geriatric neurorehabilitation [22], with qualitative studies highlighting improved patient satisfaction through shared decision-making and holistic goal setting [22]. Qualitative studies further highlight improved patient satisfaction through these shared decision-making processes and holistic goal setting. For instance, in community-based settings, interdisciplinary teams facilitate flexible role-sharing, leading to better functional outcomes and reduced caregiver strain in older adults [22,58]. This collaborative model, as illustrated in Figure 3, is particularly effective in addressing biopsychosocial aspects, ensuring patient-centered care.

Emotional support and QOL

Physical activity and exercise enhance motor and cognitive functions, foster autonomy in daily tasks, and reduce the risk of stroke recurrence, offering significant benefits for individuals with neurological conditions [48,59]. Despite these advantages, physical inactivity remains prevalent post-stroke, with motivation emerging as a critical determinant in sustaining engagement in therapeutic activities [48,59]. Employing motivational techniques can significantly boost exercise adherence, promote long-term behavior change, enhance self-confidence, and improve functional outcomes in individuals with chronic neurological disorders [60,61].

Therapeutic Relationship and Motivation

Physical therapists employ evidence-based motivational techniques to enhance patient engagement in rehabilitation, fostering a supportive environment through active listening, clear communication, and patient-centered approaches. These strategies help establish a therapeutic rapport that strengthens treatment compliance and improves overall outcomes [62,63]. Collaborative goal-setting, involving patients, their families, and interdisciplinary teams, creates achievable, measurable objectives that bolster self-efficacy. Recognizing progress milestones, regardless of scale, reinforces sustained participation and cultivates confidence in the rehabilitation process [63].

Personalized intervention approaches are tailored to align with individual capabilities, preferences, and functional priorities, ensuring a customized therapeutic experience. Progressive activity modification ensures appropriate challenge levels while maintaining patient safety and engagement throughout the rehabilitation [63]. Patient education regarding neurological conditions, rehabilitation benefits, and long-term management strategies promotes informed participation in treatment decisions and continued engagement in home-based activities [63]. Key strategies include active listening (used by 94% of therapists) and sharing success stories, which can increase adherence by 20-30% in stroke survivors [64]. Delphi studies identify 19 effective techniques, such as praise and goal-oriented practice, tailored to neurological contexts to foster intrinsic motivation [65].

QOL Considerations

QOL refers to physical, psychological, social, and functional areas that are significantly influenced by neurological diseases. PT methods directly address numerous QOL variables by improving mobility, reducing discomfort, increasing independence, and engaging in meaningful activities [66-68]. The restoration of functional abilities for ADL is closely linked to improved QOL measures and a reduced caregiver burden among older adults with neurological conditions, highlighting the practical benefits of targeted rehabilitation efforts [66].

Social engagement plays a vital role in well-being, and PT interventions that focus on mobility and functional capacity allow people to continue participating in social activities, retain vital relationships, and interact with the community, all of which are crucial for their psychological health [68]. The comprehensive approach of PT, which integrates functional restoration with psychosocial support, significantly contributes to maintaining QOL and promoting successful aging in individuals with neurological conditions, offering a balanced strategy to address both physical and emotional needs [68].

Future trends in neurorehabilitation

Emerging technologies offer significant opportunities to enhance PT interventions for older individuals with neurological disorders [69]. Brain-computer interfaces (BCIs) represent a promising innovation, converting neural signals that reflect movement intentions into functional feedback, particularly benefiting patients unable to engage in conventional PT methods. Although clinical interest in BCI technology is increasing, operational challenges, such as technical limitations and integration issues, currently hinder its widespread adoption in rehabilitation settings [70].

BCIs present promising solutions for patients with severe motor impairments by circumventing damaged neural pathways, facilitating early rehabilitation interventions. These systems translate brain signals into control commands for assistive devices, offering potential restoration of functional capabilities in individuals affected by strokes, traumatic brain injuries, and spinal cord injuries [71]. Neuromodulation techniques, including transcranial magnetic stimulation and other neurostimulation approaches, show promise as adjunctive treatments to traditional PT interventions. These techniques may improve neuroplasticity and motor learning when integrated into evidence-based rehabilitation protocols [69].

Robot-assisted rehabilitation involving advanced robotics and exoskeletons allows for intensive and repetitive training while lowering the therapist’s workload and enabling precise movement control. Incorporating these technologies into traditional PT procedures may improve functional outcomes in neurological rehabilitation [72]. Preliminary clinical investigations provide promising early evidence for these innovations. For example, pilot trials of exoskeleton-assisted gait training in older adults and neurological populations have reported significant gains in walking endurance, mobility, and engagement [71,72]. Similarly, BCI-based rehabilitation has demonstrated the ability to promote cortical reorganization and improve motor recovery in chronic stroke patients [71]. These findings highlight the potential benefits of integrating emerging technologies into conventional PT programs [69]. Implementation of emerging technologies requires consideration of cost-effectiveness, safety profiles, training requirements, and integration with existing rehabilitation frameworks. Future developments must target the scalability and accessibility of these models to ensure their widespread clinical adoption [69].

Telerehabilitation, enhanced by wearables and virtual reality, has expanded access during pandemics, improving balance and gait in home settings with high patient satisfaction. Exoskeletons like Ekso GT enable overground walking, promoting neuroplasticity in traumatic brain injury and stroke patients, with studies showing 50-100% increases in step counts per session. These technologies address barriers like therapist burden and geographic limitations, paving the way for hybrid models in geriatric care [73]. Though emerging technologies demonstrate transformative potential, scalability, ethical considerations, and cost-effectiveness parameters must be addressed to enhance the sustainable implementation in clinical practices. The ethical challenges that are mostly encountered consist of data privacy, overreliance on technology, and obtaining informed consent from patients before the application of technologies [69,70]. Additionally, the cost-effectiveness analysis is also vital, with preliminary data illustrating that the initial cost of technology applications provides long-term savings through reduced therapist hours and improved outcomes. However, equitable reimbursement models and value-based care frameworks are essential to democratize access [69,72]. Moreover, another barrier observed is scalability, as high-tech technologies require training and infrastructure, thereby challenging utilization in resource-constrained settings; however, combining telerehabilitation with wearables facilitates broader dissemination [73].

## Conclusions

PT plays a crucial role in managing neurological changes associated with aging by targeting functional impairments through evidence-based interventions that significantly improve mobility, strength, balance, and independence in older adults and thereby reducing impaired balance and enhancing strength in muscles. As the aging population continues to grow, the demand for specialized neurological PT services will also increase correspondingly. Targeted interventions, such as gait training, balance exercises, strength training, and functional mobility activities, can significantly enhance outcomes and QOL in this population. Moreover, resistance intervention reduces oxidative stress and facilitates mitochondrial function. Multicomponent protocols reinforce postural equilibrium and dual-task proficiency, and neuromodulatory exercises stimulate BDNF. PT alleviates motor deficits, induces neural plasticity, and averts sequelae like recurrent falls, frailty escalation, and institutional dependency, with amplified impact through interdisciplinary synergies involving occupational adaptations for daily living, speech-language remediation for dysphagia, and unified goal-setting to optimize biopsychosocial outcomes. However, effective management necessitates thorough assessment, customized treatment planning, and interdisciplinary collaboration. The limitations observed in the current review involved literature search on limited databases, inclusion of only English language text, and absence of systematic review and meta-analysis of the included articles, as it is a narrative review, underscoring opportunities for future scope. As the field evolves, maintaining focus on evidence-based practice while embracing technological advances will ensure optimal outcomes for older adults aging with neurological conditions, with the growing body of supporting evidence underscoring the profession’s critical role in promoting successful aging and functional independence throughout life.
